# Post-conflict development, reviewing the water sector in Somalia

**DOI:** 10.1007/s10668-021-02096-3

**Published:** 2022-01-03

**Authors:** Khaldoon A. Mourad

**Affiliations:** 1 Swedish National Road and Transport Research Institute (VTI), 583 30 Linköping, Sweden; 2 Lund University and the Centre for Sustainable Visions, Lund, Sweden

**Keywords:** Water governance, Investment, Capacity building, Resilience, Climate change, Corruption

## Abstract

Somali post-conflict development faces many challenges that affect the sustainability of the water sector. This paper reviews and analyses the post-conflict development activities in the water sector through local communications and reviewing published materials and databases from international players in Somalia, funding agencies and financial tracking service. The paper has shown that there has been great attention and support given to the country during its post-conflict development. However, most of these initiatives and projects have focused on emerging issues such as tackling food security and water, sanitation and hygiene services. The paper also shows that the continuous funding of emerging issues in Somalia has reduced its long-term sustainability of the water sector and limited its national and long-term benefits but has increased corruption due to increase the gap between actors and local people. Therefore, new transparent cooperative initiatives are needed based on transparent involvement and coordination among donors, local authorities and implementers to improve and develop the water sector and the livelihood in Somalia through a solid water governance system.

## Introduction

### Background

**Fig. 1:**
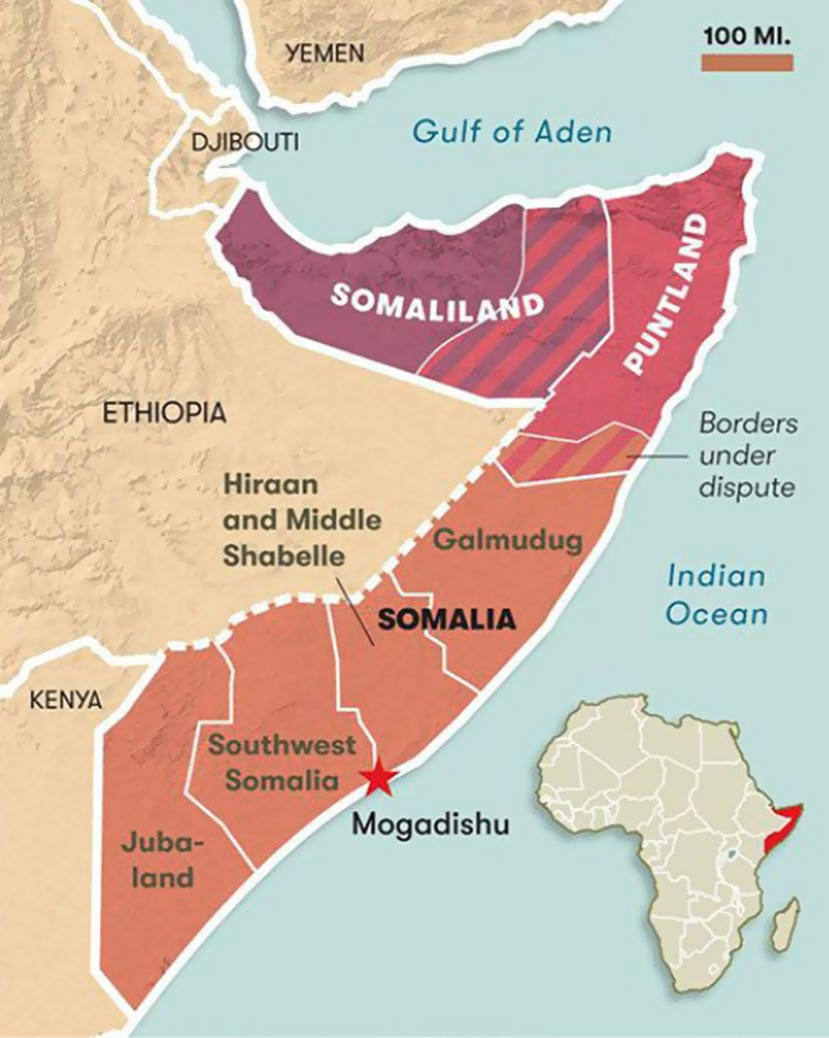
Somalia and its states (Magazine and American at War 2020)

The federal republic of Somalia has a total population of 15 million on an area of 637,655 Km^2^ and is surrounded by the Indian Ocean, Kenya, Ethiopia and Djibouti (Fig. 1). The country has five federal states (Puntland, Galmudug, Hirshabelle, Jubaland and South West State), excluding Somaliland, which declared its independency after the fall of Somalia’s military dictator Muhammad Siad Barre in 1991 (Balthasar, 2014).

The civil war that started after the collapse of the central government of Somalia in 1991 affected all aspects of life in Somalia and destroyed most of the water systems (Jama & Mourad, 2019), which has affected water resources management and created a poor governance system. After the years of the civil war, the federal states tried to improve the situation with the help of the international community. However, the administrations of the federal member states couldn't improve the livelihood of the Somali people (Balthasar, 2014) due to the lack of a documented system of roles and responsibilities, which by its turn, increased the gaps between the different institutions, reduced the level of collaboration to solve water challenges, reduced transparency and increased the competition to attract funders (Jama & Mourad, 2019; Mourad, 2020).

In addition to the civil war, Somalia has gone through many disasters. The cholera outbreak of the year 2000 killed hundreds of Somalis due to unsanitary water; due to drought, famine, and fighting of the year 2006, thousands of Somalis fled to Kenya, Al-Shabab (an alliance with al-Qaeda) concentrated its troops for offensives, and in the same year famine killed about 260,000 people (Jama & Mourad, 2019). All the above-mentioned challenges affected drinking water affordability and most of the Water, Sanitation and Hygiene (WASH) services in Somalia (Abdulle & Gillian, 2012).

### Water and life

Surface water resources in Somalia come mainly from two rivers Juba and Shabelle that originate from the Ethiopian highlands the flowing to the Indian Ocean at the southern part of Somalia (Sebhat, 2014). The lack of transboundary water agreement between the neighbouring countries treaty affected the possibility of constructing all proposed water infrastructure project including the Baardheere Dam (Mourad, 2020; Salman, 2011), which makes the rivers vulnerable to upstream uses. The average flows of Juba and Shabelle rivers on the Ethiopian border are about 186 m^3^/s and 75 m^3^/s, respectively (Sebhat, 2014).

According to Food and Agriculture Organization—Somalia Water and Land Information Management (FAO-SWALIM), the Juba and Shabelle rivers face many challenges including:

- Flooding is a major risk in the river basins;
- Insecurity affected the development of services in many areas;
- Lack of data, monitoring and information management of the water sector;
- Lack of sustainable development of the transboundary river basins; and
- Lack of resources to develop and implement water management plans.

In addition to the two rivers, Somalia has about 250 dams. The United Nations Development Programme (UNDP) supported the construction and repair of 5 earth and 3 sand dams in Puntland in 2017 and 2018 (Reliefweb, 2020). Small earth dams are needed across the country to support the livestock sector. However, using the same water resource for domestic and livestock uses poses health risks for the spread of water-borne diseases.

Groundwaters play an important role in ensuring water security for people and livestock. GW includes 823 boreholes, 1700 shallow wells and 352 springs. The average annual GW from boreholes and wells can be estimated at 0.25 km^3^, no estimates were found regarding the springs (IOM, 2013). GW face two main challenges overexploitation and contamination. The majority of GW sources in the country have high salinity levels. The Total Dissolved Solids (TDS) range is 637 to 2,070 mg/l, which is over the required standard for drinking water at 500 mg/l according to World Health Organization (WHO) (IOM, 2013). Moreover, many shallow wells are unprotected, which makes them vulnerable to contamination*.*

Groundwater recharge is estimated at 3 to 5% of annual rainfall (Plac, 2001). However, artificial recharge, the process whereby surplus surface water is transferred underground to be stored in an aquifer for later abstraction and use, is important and can cover maintain groundwater resources and improve its quality, if planned and implemented in a proper way. In general, groundwater resources face two major problems overexploitation and pollution due to the poor governance system, which doesn’t have proper control and monitoring mechanisms (FAO, 2013). In this regard, people kept using polluted groundwater for domestic and agriculture uses, which has increased water-borne diseases among Somalians in the rural areas (Khan, 2018).

The vast poverty and the absence of proper drinking water systems and authorities opened the doors to the Public–Private-Partnerships (PPPs), who are the main drinking water provider in many areas in spite of the high prices and the low quality (Jama & Mourad, 2019). According to the recent Somalia WASH cluster dashboard, two million of Somalis are in need of proper WASH services (Mourad, 2020; Reliefweb, 2020). Moreover, the years of war created inequalities between men and women from the first hand and between men themselves on the second hand (Rift Valley Institute, 2017). Moreover, the high-water prices, more than 1 USD per cubic meter, and the long distances to get water are the main problems facing poor families, especially women. In Somalia, 0% of the used water is reused, wastewater in coastal areas goes to the sea and in other areas discharged to uncontrolled septic tanks. Moreover, on-site sanitation practices such as pit latrines and shallow underground tanks that are widely used in Somalia are intensively polluting the groundwater resources. Moreover, Open defecation rates are high and a proper handwashing practices are very low.

Furthermore, insecurity poses challenges to goals of stability and development in Somalia. Many clans tried to control some pasture areas and boreholes using their power, which increased the level on conflicts in some areas in 2010 and 2011 (PRI, 2014; The New Humanitarian, 2010).

Last but not least, Floods and droughts are threatening the country causing displacement, destroying agriculture production and spreading water-borne diseases (WB, 2018a). According to the World Bank (WB, 2019a), 6.3 million Somali had suffered from acute hunger by 2017. Therefore, the United Nations Children's Fund (UNICEF), the World Bank (WB) and FAO focus on supporting monitoring and resilience to reduce negative impacts of these climatic disasters and to support livelihood and livestock in rural areas (FAO, 2017; UNICEF, 2018a; WB, 2018b; WHO, 2019).

Water demand is divided into three main sectors: (1) domestic demand that was estimated at 20 Litres per Capita per Day (Lpcpd) in rural areas and 50 Lpcpdin urban areas (Sebhat, 2015), which represents 0.2 Million Cubic Meter (MCM).; Agriculture demand represents 98% of the total demand (FAO); and (3) Livestock demand: Water demand for cattle, sheep/goats and camels were assumed as 25, 1.6 and 12 L per head per day (l/h/d) (Michalscheck et al., 2015). According to the Somalia Livestock Sector Development Strategy (LSDS), Somalia has 6,647,164, 5,530,921, 12,983,154 and 30,516,421 camel, cattle, sheep and goat, respectively. Therefore, the annual water livestock demand was estimated at 110 MCM.

### Objective and the significance of the study

More than 50% of the population in Somalia doesn’t have access to improved water sources, which has increased the spread of water-borne diseases. According to FAO-AQUASTAT, the total available renewable water resources are way far from the total withdrawal, which indicated that Somalia doesn't face water scarcity, Somalia poor water management (Mourad, 2020). This has created water deficits over the country. Therefore, an improved management system can solve most of the water challenges in Somalia.

The civil war destroyed the water systems and posed many challenges for its development to achieve Sustainable Development Goals (Jama & Mourad, 2019). Moreover, the upstream developments in Ethiopia have affected water availability and irrigation practices in Somalia (Michalscheck et al., 2015). These challenges cover many areas, as described above, starting from climatic threats (floods and droughts) ending with small water management issues such as the access of livestock to the same water points that humans use, which has increased health vulnerabilities in many rural areas. All these challenges need to be addressed, which encourage the local authorities to seek funding and encouraged the international communities to play a role in the country’s development. However, the lack of cooperation and coordination between these different stakeholders couldn’t help the country to overcome all of these challenges (Balthasar, 2014; Mourad, 2020).

Capobianco and Naidu (Capobianco & Naidu, 2011) found, in their analyses of the aids that were given to the health sector in Somalia between 2000 and 2009, that mistakes and lack of coordination among donors, local authorities and implementers were the results of one decade in the health sector in Somalia.

Therefore, and due to the need for transparent development of the water sector in Somalia, this paper aims to assess, analyse and understand the post-conflict development status in Somalia focusing on most current funding projects across sub-sectors of the water sector.

## Methodology

The paper is based on a mission to analyse the water sector situation in Somalia with a full support from local authorities and international partners. Qualitative methods were involved to collect and analyse the needed data in this paper. Data were collected through:

- Structured interviews and semi-structured interviews were performed via emails, phone calls and WhatsApp, during February–April 2020. At this step, national and international partners who are working in water projects in Somalia were contacted and they shared most of their past and ongoing activities as documents and websites. The communication involved the following national organizations: Ministry of Agriculture (MoA), Ministry of Energy and Water Resources (MoEWR), MoE, & OPM; and the following international partners: UNDP, FAO, UNICEF, International Organization for Migration (IOM) and the World bank.
- Other materials were found at the Financial Tracking Services, https://fts.unocha.org
- Moreover, in March 2020, I with the MoEWR and other national and international partners, organized and hold the National Technical Group workshop to analyse the challenges of the water sector and to start working to develop the national water compact (Mourad, 2020). The outcomes of this workshop were also used in this paper.

## Data analysis of funding initiatives by sector

According to the Humanitarian response plan, in 2020, Somalia requires 1,05 billion USD, and to date, Somalia has received 127,1 Million USD.

In this paper, the current investment in the water sector in Somalia will be discussed covering four areas:

1. WASH service,
2. Agriculture and Livestock,
3. Resilience and climate change, and.
4. Capacity building and water management.

Based on the Financial Tracking Services, https://fts.unocha.org, Table 1 summarizes the countries received funds over the last 15 years (FTS 2020) and shows that the food security and WASH sectors received about 43% and 5% of the total funds, respectively.

**Table 1:** Received funds from 2007–2020

Year	Total fund Million USD	WASH sector share (Million USD)	Agriculture & food security (Million USD)
2020	127.1	1.9 (until March only)	49.2
2019	899.3	29.93	362.9
2018	925.98	41.05	372.7
2017	1038	60.67	438
2016	498.57	24.17	189.2
2015	385.99	23.19	140.2
2014	457.9	25.03	177.5
2013	586.13	43.34	218.4
2012	657.3	28.4	349.1
2011	879.9	59	440.5
2010	440.1	23.248	257.6
2009	663.8	20.5	375.9
2008	640.1	20.56	360
2007	363.4	10.5	160.2
Total	6611.19	338.608	3106.6

### WASH services

The civil war, poverty, the absence of the governmental role and the lack of water services have increased the feeling of water scarcity and opened the doors for Public–Private Partnerships (PPP), which are the main water provider in many areas in Somali. However, there are many problems needed to be solved, mainly water price and quality (Jama & Mourad, 2019).

Population growth and urbanization are challenges for the improvement of the WASH sector, and other challenges include: a high rate of failure of rural and urban water supplies; open defecation; the limited human and financial resources in the WASH sector (which led to the poor sustainability of WASH initiatives); the lack of proper WASH facilities in schools (which led to high dropouts among girls); and many rural areas lacking proper WASH services. And so, in general, access to improved water and sanitation facilities in Somalia stands at 45% and 25%, respectively (Mourad, 2020).

**Fig. 2:**
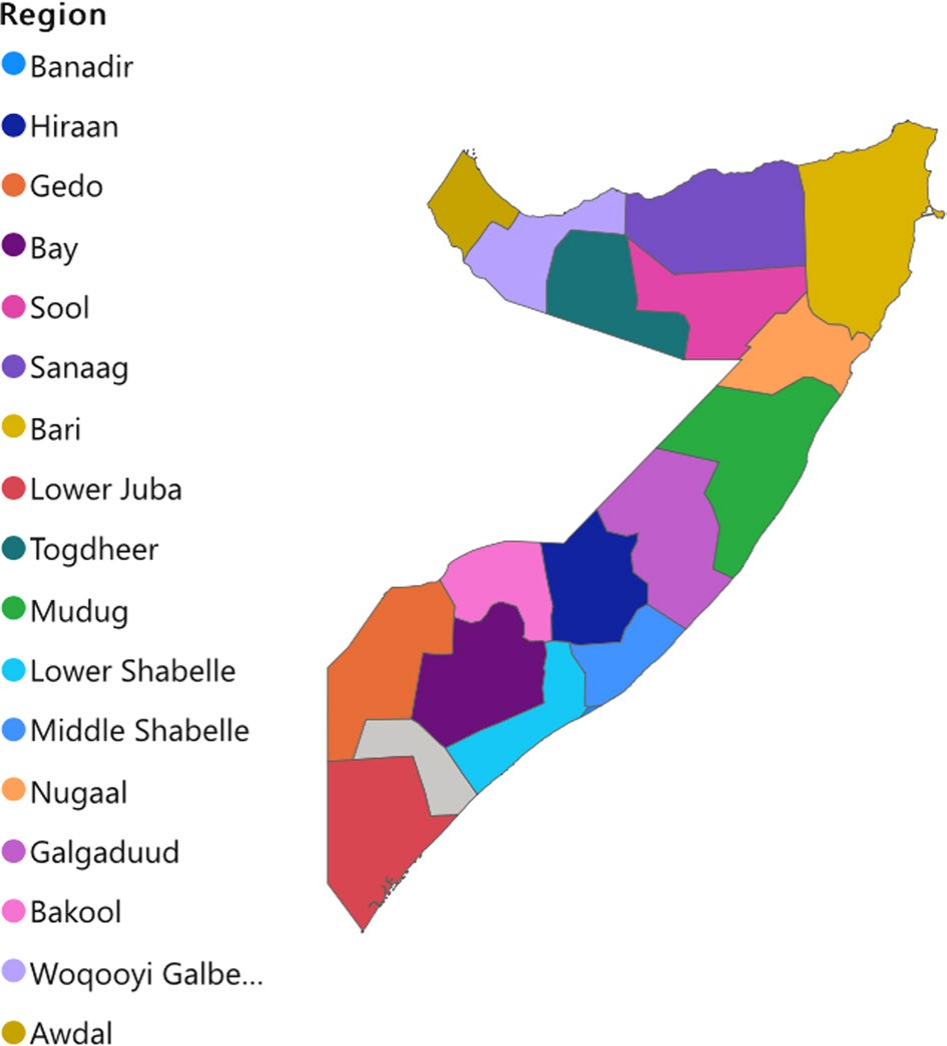
WASH cluster’s operational research and presence map in Somalia

The Somalia Wash cluster is coordinated by UNICEF with 46 active partners. It brings together local NGOs, International NGOs, Government departments and UN agencies that are all involved in the implementation of WASH activities in Somalia. Its main aim is to provide the Somali people with WASH services. The WASH cluster projects are funded by Somalia Humanitarian Fund (SHF), EC, UNICEF, USA, UK, Central Emergency Response Fund (CERF) and Intergovernmental Authority on Development (IGAD). Figure 2 presents the operation and presence map of the WASH cluster.

During the past years, Somalia has witnessed a great improvement in WASH services. However, many people still lack WASH services. The UNICEF, in its global strategy, aims to achieve access to adequate and equitable drinking water, sanitation and hygiene for all (UNICEF, 2020a).

**Fig. 3:**
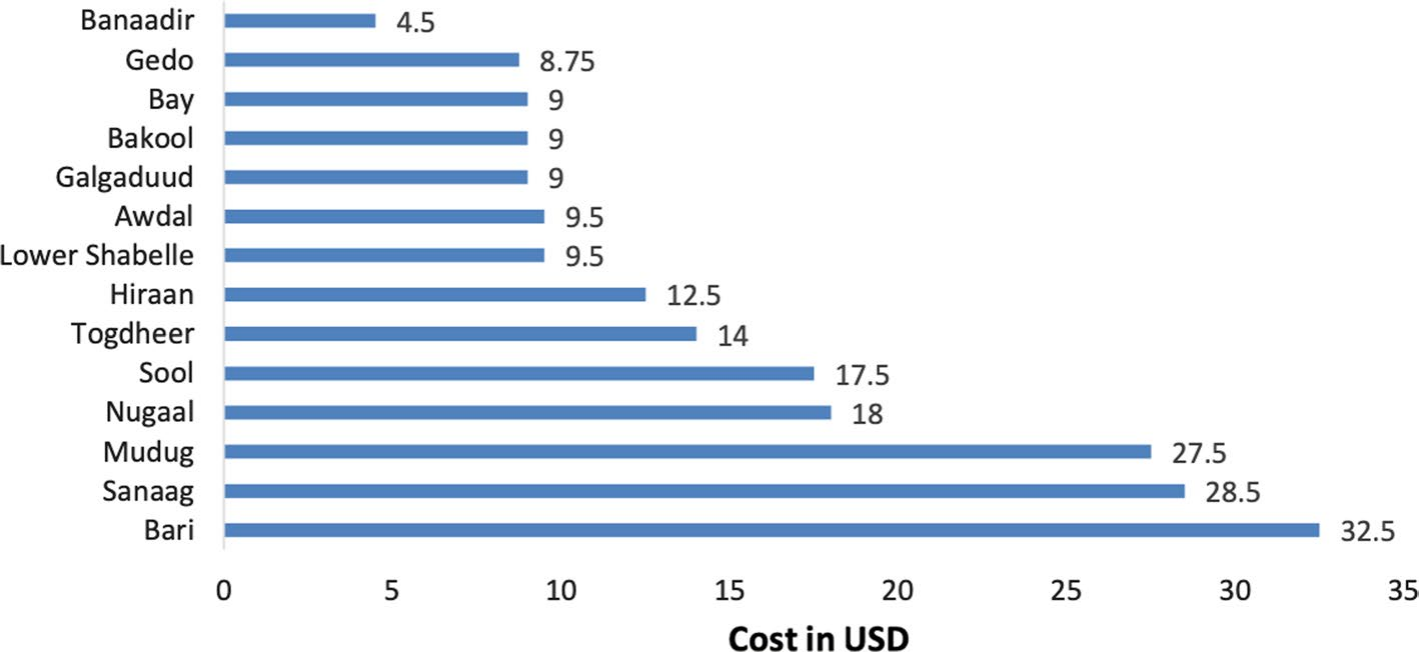
Water prices for one cubic meter in some areas in Somalia

Moreover, to address water scarcity during the dry periods, UNICEF and other donors have used water trucking/vouchers to supply the affected population with drinking water. However, it was found that water trucking is the most expensive option for the provision of water supply. However, rehabilitating the existing water systems can help to reduce these costs by 80%. The South-Central areas are served by shallow well-based water supply systems, which makes the prices lower than deep boreholes (200–400 m) and mechanized water supply systems (UNICEF, 2019). Figure 3 represents water prices in 2018–2019 throughout the different regions (UNICEF, 2019).

The Somalia WASH Cluster publishes monthly updates for the improvement in WASH services in Somalia. The recent Dashboard was published on the 31st of March 2020 (Table 2) (Somalia, 2020).

**Table 2:** WASH services coverage during January March 2020

The number of people reached with access to sustainable water services since January 2020	168 407
The number of people reached with access to temporary safe water since January 2020	349 170
The number of people reached with access to sanitation since January 2020	66 520
The number of people reached with hygiene promotion activities since January 2020	226 773

#### Somalia infrastructure fund (SIF)

The SIF is managed and administrated by the African Development Bank (AfDB) in Kenya and financed by AfDB, UK Aid, Italy, the Islamic Development Bank (IsDB) and the EU (AfDB, 2020).

The most important ongoing projects are:

- Improving Access to water and sanitation services (IAWSS) ($11 million: 2016–2020) at Jubbaland, Southwest, Galmudug, Puntland and HirShabelle. The executor is the MoEWR, while the implementer is the IOM. The project has three main components capacity development, infrastructure and project management of the project focuses on improving water systems, sanitation services and rainwater harvesting (AfDB, 2020).
- In 2019, the AfDB signed a 28.8 million USD grant focusing on road and water projects. The Kismayo-Baidoa Urban Water Supply and Sanitation Project ($12 million: 2019–2023) aims to increase access to WASH services and capacity development to improve the services in the towns of Kismayo (Jubbaland state) and Baidoa (South West state). The direct beneficiaries are estimated to be 200,000 people (AfDB, 2019).

#### Other projects

Funded by Germany.

- World Vision International (WVI) is running a 984,089 US$ project to Improve access to food, water, sanitation, health care and nutrition by providing essential goods, services and cash support to vulnerable households of internally displaced people and host communities in Baidoa.
- Save the Children (SC) is running a US$ 5.5 Million project to improve access to health, nutrition, WASH, education, child protection and cash benefits for vulnerable children and their families in Somalia
  Funded by Japan.
- UNICEF received 118 000 US$ for Emergency Water Supply, Sanitation and Hygiene interventions for emergency affected and vulnerable populations throughout Somalia.

Moreover, there are three incoming projects in 2020 covering the WASH sector (Table 3).

**Table 3:** In coming funding covering WASH in 2020

Funding source	Acting organization	Amount US$	Project title
Private	WVI	262,500	Improving WASH service delivery for disaster-affected communities in Somalia
Japan	UNICEF	181,818	Emergency Water Supply, Sanitation and Hygiene interventions for emergency affected and vulnerable populations in Somalia
Japan	IOM	1,459,752	Provision of lifesaving assistance and sustainable solution of safe water sanitation and hygiene (WASH) services, for people in crises and their host communities in multiple regions in Somalia

### Agriculture and livestock

Agriculture and livestock are considered the main contributors to the Somalia’s Gross Domestic Product (GDP). The shares of livestock and crop production of Somalia’s GDP were estimated at 60.7% and 10% in 2016, respectively. Livestock's share of Somalia’s GDP was estimated at 60.7% in 2016. Somalia approached self-sufficiency in cereals before the civil war. However, recently it provided 22% on average of the country’s per capita cereal needs (Drought Impact Needs Assessment (DINA) 2018). In 2017, Somalia's exports were estimated at 198 million USD, about half the 2012 estimates, Somalia’s exported commodities include sheep, goats, camels, bananas, hides, fish, charcoal and scrap metal. Somalia’s imports were estimated at 2. billion USD, more than double the 2012 estimates. Imported commodities include raw sugar, rice, manufactures, petroleum products, foodstuffs and construction materials (OEC, 2017). The annual decrease in exports at 13%, and the annual increase in imports at 16%, pose a challenge to development and poverty reduction in Somalia.

The agriculture sector is covered within the food security cluster, which aims to be the primary source of information on the humanitarian response by addressing food insecurity, and by providing its members with a strategic vision and guidance in their response to the acute and underlying causes of the crisis. It has six working groups: COVID-19 TWG; Cash and Markets; Inter-Cluster Nutrition; Food Security and Livelihoods in Urban Settings; Preparedness and Resilience; and Programme Quality.

FAO is the most important organization that receives funds and delivers agricultural projects in Somalia. *‘Addressing acute food insecurity in rural areas of* Somalia’ is their projects mission, and for them to execute this, FAO received 7 million USD from United States Agency for International Development (USAID) to launch several activities in 2019, which created a second phase of the project in 2020 focusing on resilience programs to support rural communities (FAO, 2020).

Within its project *’Support to the Food Security and Nutrition Analysis Unit (FSNAU) for Somalia'* and funded by Switzerland, FAO will give support food security, resilience and SWALIM.

Moreover, within its project, ‘Addressing Acute Food Insecurity in Rural Somalia’, FAO will run three projects throughout 2020 (Table 4).

**Table 4:** Incoming FAO projects in 2020. (Source: FTS 2020: https://fts.unocha.org/appeals/831/flows?boundary=incoming)

Funding source	Acting organization	Amount US$	Description
USA	FAO	22,000,000	Improving and sustaining food security in rural Somalia
Sweden	FAO	3,060,912	Increase the resilience of livelihoods to threats and crises
Germany	FAO	3,657,504	Target desert-locust-affected areas through interventions and efforts to protect the livelihoods of desert-locust-affected farmers, herders and agropastoralists

For the livestock sector, FAO is running five Technical cooperation programs (Table 5) (FAO, 2020).

**Table 5:** FAO technical cooperation programme in Somalia

Title	Years	Budget (US$)
Assistance for capacity development in desert locust control in Somalia	2020–2021	500,000
Strengthening capacity of the Ministry of Fisheries and Marine Resources in Somalia for effective monitoring of licensed foreign fishing vessels	2019–2021	178,000
Improving the coordination and capacity of Ministry of Livestock, Forestry and Range and other Somali institutions to control trade limiting livestock diseases	2019–2020	364,000
Mapping and development of a strategy for the management of alien invasive insect pests of Agricultural crops in Somalia	2019–2020	166,000
Forestry and Range to respond to Drought emergency	2019–2020	500,000

### Resilience and climate change

Before the civil war, there was a system of canals that channelled water from the Shabelle to irrigate the surrounding farmland. Rehabilitating these canals may be expensive, but through a governmental project financed by some international partners, it can be a good alternative for flood resilience in the Shabelle river.

The Juba and Shabelle rivers experience great seasonal variation inflows but never dry up completely. Moreover, there are a number of seasonal rivers, which flow only during the rainy season, and after heavy storms the flow is often accompanied by flash floods. The Juba and Shabelle rivers have two peak flows during the rainy seasons, which occur in October and September respectively (Michalscheck et al., 2015).

According to the Somalia Drought Impact and Needs Assessment (Drought Impact Needs Assessment (DINA) 2018), due to the drought of 2016–2017, the Shabelle River dried out completely, which affected water quality and quantity and increased water-related risks. This highlighted the weaknesses of water management practices regarding testing, monitoring, regulation and water protection.

Therefore, many funding organizations started initiatives to support Somalia in facing climatic challenges (floods and droughts). The following are some of the ongoing projects:

#### UNDP

- A project of US$21 million (2015–2022) to enhance climate change resilience. This project aims at (1) supporting ministries, districts, Non-Governmental Organizations (NGOs), Community-Based Organizations (CBOs) aiming at reducing climatic risks by including disaster preparedness in natural resource management practices, (2) supporting community-led activities (capturing water using small scale infrastructure) and (3) reducing flood impacts by using water diversion techniques and reforestation (UNDP, 2020a).
- Support for Integrated Water Resources Management (IWRM) (US$87.575 million: 2019–2023) aiming at increasing water access and decreasing water-related disasters that face Somali pastoralists focusing on four sectors: policy reform, capacity building, improved water monitoring and using best IWRM practices (UNDP, 2019a).
- In November 2019, Somalia and UNDP launched a US$10 million project (US$8.5 million from GEF and US$1.5 million from UNDP) to support rural communities with access to water resources through a proper water management plan and early-warning systems. The project is led by DECC and implemented by the MoEWR. The minister of the MoEWR, H.E. Fowzia Mohammed Sheikh highlighted the importance of this project to improve climate change resilience of water authorities in Somalia (UNDP, 2019b).

#### WB

The World Bank started the ‘*Somalia—Water for Agro-pastoral Productivity and Resilience (US$42 million: 2019–2023)’.* The project aims to develop water and agricultural services among agro-pastoralist communities in dryland areas of Somalia through (1) improving access to and management of water resources; (2) supporting improved management of rangelands and forests; and 3) strengthening the agricultural knowledge and innovation systems (WB, 2018c).

#### WVI

Funded by seven donors (EU, FAO, USAID, Swiss Agency for Development, Danish International Development Agency (DANIDA), Sweden and Australia), The Somalia Resilience Program (SomReP) was started in 2014 and focuses on building resilience to drought and related risks in pastoralist, agro-pastoralist and peri-urban households and communities in Somalia. The project is implemented by a consortium of international NGOs led by World Vision. The SomReP Phase II (2018–2023) focuses on ensuring that food security system and the water ecosystem are robust and resilient. The target areas are Somaliland and Puntland.

#### Cooperation International (COOPI)

As part of the SomRep project, COOPI is implementing the Department of Foreign Affairs and Trade, Australia (DFAT 6) project. The project aims at: improving disaster risk governance, maintaining the ecosystems; capacity development for youth, and promoting small business activities (COOPI, 2019).

#### Sweden

The strategy for Sweden’s development cooperation with Somalia (US$303 million: 2018–2022) focuses on peace, resilience, human rights, gender equality, and sustainable development (GoS, 2018).

**SDRI:** Somali Disaster Resilience Institute.

SDRI, http://sdri.so/services-sdri/, has three projects focusing on resilience, two of them are funded by USAID, while the third is funded by Bill and Melinda Gates Foundation.

#### FAO

FAO has many ongoing projects within food security and resilience, and most of them has started in 2018–2019 with a total fund about US$100 million.

### Capacity building and water management

The lack of skilled workers has affected the construction, management and maintenance of the water supply systems (UNICEF, 2018b).

Capacity development is needed in Somalia to:

- Advance national and federal states’ organizational visions in the integration of good water governance.
- Foster the abilities of individuals, institutions and communities to overcome challenges and to contribute towards local solutions in the water sector and natural resources management.

The following are some of the ongoing projects:

#### ADF

The African Development Fund (ADF) is financing: Economic & Financial Governance Institutional Support Project Phase I (2017–2021). The project aims to strengthen the institutional capacity of core departments charged with financial governance responsibilities in the Ministry of Finance, the Office of the Auditor General and in the infrastructure line Ministries of Energy and Water Resources, Transport & Civil Aviation, and Ports & Marine Transport (AfDB, 2017).

#### AfDB

The first component of the AfDB project, presented in Table 4 (Improving Access to Water and Sanitation Services in Somalia), aims at improving the policy environment of the WASH sector and the rehabilitation of the MoEWR buildings at the Federal Member States.

#### FAO

Funded by EU, FAO is running the Integrated Land and Water Resources Management (ILWRM) project focusing on building the capacity of governmental staff in Somaliland and Puntland in the area of (ILWRM) (EU, 2019).

#### The World Bank (WB)

The World Bank started the Somalia Urban Resilience Project (SURP) ($9 million: 2018–20,219), which created about 5000 person-days of employment by 2019 (SURP, 2020). Based on the gains of the existing Somalia Urban Resilience Project (SURP), the WB approved $112 million to finance the Somalia Urban Resilience Project II (SURP II) (2020–2024), which aims to strengthen local government capacity and provide urban infrastructure for Somalis (WB, 2019b). Moreover, the WB has increased the support to Somalia toward economic and social recovery (WB, 2020).

#### UNDP

In March 2020, UNDP completed a five-year project for institutional capacity development ($28.84 million: 2015–2020) focusing on youth, business development, agriculture and water sectors. Moreover, in 2017, UNDP in collaboration with the Federal Government of Somalia, MoPIED and MoTI, launched the “Innovate for Somalia” to help youth start their business (UNDP, 2020b).

## Discussion

This paper aims to assess and understand the post-conflict development status in Somalia focusing on most current funding projects across sub-sectors of the water sector. However, some investment initiatives have not been covered by any of the national or international datasets, which has made it difficult to assess its importance or to analyse it in this paper. The tables in Appendix section show that there are much funding and implementing partners all over the country. However, the levels of transparency, cooperation and coordination are missing, which increases the risks of overlapping and corruption.

The destroyed water services led to a cholera outbreak which occurred in 2000 then drought, famine and fighting in 2006 (TWR, 2011). Worse came in 2010 when Al-Shabab group began a campaign to capture the capital (Atlantic and Timeline: Somalia, 1991–2008). All these factors affected free access to WASH services, water iniquity and security (Abdulle & Gillian, 2012). Therefore, many international organizations independently started trying to support the country to overcome this miserable situation.

Post-conflict development faces three main challenges transparency, trust and corruption. In Algeria, Mourad and Avery (Mourad & Avery, 2019) found that many of the post-conflict development projects were satisfactorily implemented and most of the loans were closed from 7 to 27 months before the end of their proposed date, which highlighted a very important question; why to get a loan if Algeria can pay it quickly from its hydrocarbon sector? The answer was clear and reported by Andersen et al. (Andersen et al., 2020), the elite capture foreign aids in many highly aid-dependent countries, which is applied to Algeria and will be, for sure, applied to Somalia after shifting up from donations to loans.

On the other hand, there are ten clusters in Somalia providing the needed support and help for all areas in the country: Camp Coordination and Camp Management; Education; Emergency Shelter/Non-Fund Items (NFI); Food Security; Health; Nutrition; Protection; Child Protection; Gender Based Violence; and WASH cluster. Each cluster is coordinated by an international organization.

For the water sector, the most important clusters are WASH and food security, and the most important organizations involved in the water sector are FAO, UNICEF, WVI, WB, AfDB, UNDP, and IOM. The projects have covered all areas in Somalia with full cooperation from the Federal Government of Somalia. Analysing the funding between 2007 and 2019 has showed that Somalia has received about 7 billion USD, of which food security and the WASH sector have received about 43% and 5%, respectively, Tables 7, 8 in Appendix.

The paper showed that there are many active international partners in Somalia. Funds come from international governments, pool fund, UN agencies, inter-governmental organizations, private organizations, and NGOs. Funded projects are managed by NGOs and UN agencies. Some of the funded projects are coordinated or led by national organizations, which has increased corruption and the principle of loyalty in selecting national and international experts.

The paper also showed that within the WASH sector, UNICEF and IOM are the most active partners. IOM works with the Somali government, Somalia diaspora, civil society, and the private sectors. In 2020, UNICEF requires about 129 million USD (UNICEF, 2020b) to:

- Provide 800,000 people with emergency water services,
- Provide 220,000 people with sanitation services in vulnerable settlements and communities, and
- Provide 1,200,000 people with hygiene promotion activities and hygiene kit distribution in vulnerable settlements and communities

Within food security and resilience, FAO is the most active partner. FAO has more the 20 ongoing projects with a total budget of about 115 million US$ (Table 5). It can be seen that the most funded areas in the water sector are food security, resilience and WASH support. Most implementing partners are making great efforts in fundraising, which has helped in the continuity of most of the programs. For example, SomRep that was started in 2014, received more funds to start its phase II (2018–2023) focusing on resilience.

The water sector has received more than 3100 USD since 2007. However, improvements and long-term benefits are limited. The large possible amounts of funds made all local authorities fund-dependents. Therefore, no real work is done without funding and all governmental officials focus on bringing funds, travelling and holding lunch meetings using the international donor.

On the other hand, analyses have shown that there is a lack of international projects that focus on transboundary issues. Therefore, there is a need to establish an information-sharing system between Somalia, Ethiopia and Kenya to address these challenges and to enhance the possibility to build sustainable transboundary management plans. Bilateral institutional arrangements for cooperation between Djibouti and Somalia would facilitate the gathering of data on the groundwater resources between these two countries (Nanni, 2016).

## Conclusions

Somali post-conflict development faces many challenges: mainly a lack of trust between different actors at the national level, and the lack of resources after years of civil war. However, the willingness of international partners to help the country has made the country a theatre for international donors and projects without any real coordination between the different partners. Therefore, creating a sort of funding coordination unit or NGO can facilitate the cooperation between the different players.

Somalia is not a water-scarce country, it lacks good water governance, transparency between different stakeholders and trust between national organizations and international partner on the first hand and between the government and the local people on the second hand. The challenges that Somalia is facing are many and can be divided into three categories. Institutional, environmental and socio-economic challenges, which made the country a pool for funding and corruption. Solving these challenges needs an integrated approach focusing on long-terms goals not emergency plans.

To conclude, focusing on emergency plans have led the country to be fund-based, which reduces the long-term effects of the completed and ongoing projects. Therefore, more attention is needed for sustainable solutions, for examples rehabilitating the water system networks is a sustainable option and much economic than water trucking during drought periods. The country needs an active funding facility that can coordinate and map all projects and be the stage for proposing new projects addressing the challenges. Moreover, new transparent initiatives are needed to shift the country from donation and corruption pools, which is almost impossible under the current way of governance in Somalia.

Moreover, similar outcomes were published by Capobianco and Naidu (2011) who found that mistakes and lack of coordination among donors, local authorities and implementers were the results of one decade in the health sector in Somalia. Therefore, coordination is needed to avoid mistakes and to achieve priorities through result-oriented actions. In this regard, a Water Compact and an active water action plan (Mourad, 2020) are needed to address water challenges and solution in Somalia taking into consideration improving the livelihood of the local people and approaching the related Sustainable Development Goals SDGs.

## Appendix

See Tables

**Table 6:** Acronyms and abbreviations

AfDB	African Development Bank
CBOs	Community-Based Organizations
CERF	Central Emergency Response Fund
COOPI	Cooperation International
CW	Concern Worldwide
DANIDA	International Development Cooperation, Denmark
DINA	Drought Impact and Needs Assessment
DFAT	Department of Foreign Affairs and Trade, Australia
DRC	Danish Refugee Council
FAO	Food and Agriculture Organization
FBA	Folke Bernadotte Academy
FGS	Federal Government of Somalia
FMS	Federal Member States
GDP	Gross Domestic Product
IGAD	Intergovernmental Authority on Development
IOM	International Organization for Migration
IRW	Islamic Relief Worldwide
IsDB	Islamic Development Bank
IWRM	Integrated Water Resources Management
MoEWR	Ministry of Energy and Water Resources
PPP	Public-Private-Partnerships
RI	Relief International
SC	Save the Children
SDC	Swiss Agency for Development
SDRI	Somali Disaster Resilience Institute
SHF	Somalia Humanitarian Fund
SIDA	Swedish International Development Cooperation Agency
UNICEF	United Nations Children's Fund
UNDP	United Nations Development Programme
USAID	United States Agency for International Development
WASH	Water, Sanitation and Hygiene
WB	World Bank
WFP	World Food Programme
WVI	World Vision International

6,

**Table 7:** Received funds from 2007 to 2020

Year	Total fund Million USD	WASH sector share (Million USD)	Agriculture & food security (Million USD)
2020	127.1	1.9 (until March only)	49.2
2019	899.3	29.93	362.9
2018	925.98	41.05	372.7
2017	1038	60.67	438
2016	498.57	24.17	189.2
2015	385.99	23.19	140.2
2014	457.9	25.03	177.5
2013	586.13	43.34	218.4
2012	657.3	28.4	349.1
2011	879.9	59	440.5
2010	440.1	23.248	257.6
2009	663.8	20.5	375.9
2008	640.1	20.56	360
2007	363.4	10.5	160.2
Total	6611.19	338.608	3106.6

^7^,

**Table 8:** Incoming FAO projects in 2020

Funding source	Acting organization	Amount US$	Description
USA	FAO	22,000,000	Improving and sustaining food security in rural Somalia
Sweden	FAO	3,060,912	Increase the resilience of livelihoods to threats and crises
Germany	FAO	3,657,504	Target desert-locust-affected areas through interventions and efforts to protect the livelihoods of desert-locust-affected farmers, herders and agropastoralists

8,

**Table 9:** FAO technical cooperation programme in Somalia in livestock sector

Title	Years	Budget (US$)
Assistance for capacity development in Desert locust control in Somalia	2020–2021	500,000
Strengthening capacity of the Ministry of Fisheries and Marine Resources in Somalia for effective monitoring of licensed foreign fishing vessels	2019–2021	178,000
Improving the coordination and capacity of Ministry of Livestock, Forestry and Range and other Somali institutions to control trade limiting livestock diseases	2019–2020	364,000
Mapping and development of a strategy for the management of alien invasive insect pests of Agricultural crops in Somalia	2019–2020	166,000
Forestry and Range to respond to Drought emergency	2019–2020	500,000

^9^,

**Table 10:** Ongoing FAO projects in Somalia (food security and resilience)

Title	From	To	Budget ($)
Resilient, Inclusive and Competitive Agriculture Value Chain Development in Southern and Central Regions of Somalia	2019	2022	7,970,900
Supporting Resilient Smallholder Farming systems in Somalia	2019	2022	6,170,300
Somali Information & Resilience Building Action (SIRA)—2	2019	2022	6,315,790
Integrated Development of Food Security, Nutrition, Water and Land Analysis and Capacity for Somalia (IDACS)	2019	2021	2,144,894
Food Security and Nutrition Analysis Unit for Somalia (FSNAU)—OFDA	2019	2020	1,500,000
Addressing Acute Food Insecurity in Rural Areas of Somalia	2019	2020	14,000,000
SWALIM: Improving flood and drought risk management in Somalia	2019	2020	500,000
Addressing acute food insecurity and building resilience in rural Somalia	2019	2020	568,828
Improving and sustaining food security in rural Somalia	2019	2020	45,000,000
Early Action to Protect Livestock Assets and Food Sources in Somalia (FSNAU)	2019	2020	616,523
Programme for Somalia Water and Land Information Management (SWALIM)	2018	2021	3,382,187
Building Resilience in Middle Shabelle (BRIMS)	2018	2021	3,309,067
Support to the Food Security and Nutrition Analysis Unit (FSNAU) for Somalia	2018	2020	1,338,744
Sustained Humanitarian Assistance to Drought-affected Rural People in Somalia	2018	2020	5,415,831
Programme for Sustainable Reduction of Charcoal and Alternative Livelihoods (PROSCAL) EU	2017	2020	1,432,938
No piracy: alternatives for youth living in coastal communities of Puntland, Galmudug and Mogadishu	2016	2020	5,978,226
Integrated Land and Water Resources Management (ILWRM) project for Somaliland and Puntland	2019	2021	3,639,040
Global Network Against Food Crises Partnership Programme—Country Investment Somalia	2018	2020	4,830,918
Sustainable Charcoal Reduction and Alternative Livelihoods (PROSCAL) ITA/SWE	2017	2020	1,039,891

^10^,

**Table 11:** Improving Access to water and sanitation services in Somalia

Project title	Partners	Components	Activities
Improving Access to Water and Sanitation Services in Somalia	AfDB MoEWR UK IOM	Capacity Development	Development of WASH Strategies for MoEWR and four States Rehabilitation/upgrading of state and national water agencies buildings Procurement of equipment, vehicles and portable water quality monitoring equipment Training of key staff on water quality monitoring Travel and study tour support
		Infrastructure	Construction/rehabilitation of 20 strategic water systems, incorporating sanitation facilities, and installation of solar water pumps Construction of 53 mini solar powered/three tank water systems, with draw off and sanitation facilities Sanitation facilities and rainwater harvesting in 20 schools, markets and health institutions Mobilisation/capacity building for communities and hygiene promotion Environmental mitigation measures
		Project management	This relates to the day to day implementation of the project. It will entail the management cost of the Third-Party Implementing Agency, as well as logistics and routine project operating expenses

11,

**Table 12:** Incoming funding covering WASH in 2020

Funding source	Acting organization	Amount US$	Project title
Private	WVI	262,500	Improving WASH service delivery for disaster-affected communities in Somalia
Japan	UNICEF	181,818	Emergency Water Supply, Sanitation and Hygiene interventions for emergency affected and vulnerable populations in Somalia
Japan	IOM	1,459,752	Provision of lifesaving assistance and sustainable solution of safe water sanitation and hygiene (WASH) services, for people in crises and their host communities in multiple regions in Somalia

12.
